# Host Immunity and *Francisella tularensis*: A Review of Tularemia in Immunocompromised Patients

**DOI:** 10.3390/microorganisms9122539

**Published:** 2021-12-08

**Authors:** Olivier Bahuaud, Cécile Le Brun, Adrien Lemaignen

**Affiliations:** 1 Service de Médecine Interne et Maladies Infectieuses, Centre Hospitalier Régional Universitaire de Tours, 37000 Tours, France; olivier.bahuaud@gmail.com; 2 Faculté de Médecine, Université François Rabelais, 37000 Tours, France; 3 Service de Bactériologie-Virologie, Centre Hospitalier Régional Universitaire de Tours, 37000 Tours, France; c.lebrun@chu-tours.fr

**Keywords:** *Francisella* *tularensis*, tularemia, immunocompromised, host immunity

## Abstract

Tularemia, caused by the bacterium *Francisella tularensis,* is an infrequent zoonotic infection, well known in immunocompetent (but poorly described in immunocompromised) patients. Although there is no clear literature data about the specific characteristics of this disease in immunocompromised patients, clinical reports seem to describe a different presentation of tularemia in these patients. Moreover, atypical clinical presentations added to the fastidiousness of pathogen identification seem to be responsible for a delayed diagnosis, leading to a” loss of chance” for immunocompromised patients. In this article, we first provide an overview of the host immune responses to *Francisella* infections and discuss how immunosuppressive therapies or diseases can lead to a higher susceptibility to tularemia. Then, we describe the particular clinical patterns of tularemia in immunocompromised patients from the literature. We also provide hints of an alternative diagnostic strategy regarding these patients. In conclusion, tularemia should be considered in immunocompromised patients presenting pulmonary symptoms or unexplained fever. Molecular techniques on pathological tissues might improve diagnosis with faster results.

## 1. Introduction

Tularemia is an infrequent zoonotic disease caused by Gram negative, intracellular coccobacillus *Francisella tularensis*. It comprises three subspecies, which vary in their pathogenicity and geographic distribution.

*F. tularensis subsp. tularensis,* the most virulent one, is found mainly in North America. It has recently (sporadically) been found in Europe [1,2]. A small number of bacteria (10–50) are capable of causing severe disease in humans by the respiratory or cutaneous route, with a case-fatality rate of around 10% in the absence of specific antibiotic treatment. Due to its pathogenicity, this subspecies is considered a potential agent of bacteriological warfare [3]. F. *tularensis subsp. holarctica* is less pathogenic; it was, until 1998, the only one found in Europe, and it co-occurs in the northern USA and Canada with *F. tularensis subsp. tularensis*. The disease is identical to that induced by the previously mentioned subspecies, but less severe with a case fatality rate of 1% without treatment. *F. tularensis subsp. mediasiatica* is mainly restricted to central Asia and the south part of Russia, and is comparable to *F. tularensis subsp. holarctica* in virulence. Regardless of the subspecies, human infection either occurs through direct contact with animal reservoir, arthropods bites, indirectly through inhalation of aerosolized particles, or contact with contaminated water or soil [2,4]. 

Clinical presentation varies according to the route of entry. Ulceroglandular presentation is the predominant form of tularemia. It combines ulceration on the inoculation site of the pathogen through the skin, with a satellite inflammatory lymphadenopathy. Oculoglandular and oropharyngeal forms are clinical variants of this presentation occurring after conjunctival or pharyngeal inoculation [2]. Pneumonic tularemia results from the inhalation of contaminated aerosols and is often associated with persistent fever, coughing, and mediastinal lymph nodes, even though acute and rapidly fatal pneumonia can occur with type A strains [2,4]. Typhoidal tularemia refers to a severe systemic disease with acute fever but no symptoms of local inoculation and no evident portal of entry. This form can lead to septic shock and organ failure [5].

Diagnosis of tularemia is often delayed, as patients tend to present with mild and unspecific symptoms. Moreover, due to the fastidiousness of culture recovery of *Francisella tularensis*, diagnosis often relies on serological techniques for which results are obtained 10 to 15 days after sampling [6]. Thanks to the development of molecular techniques, quick and positive results are obtained with PCR-based methods [7,8,9]. However, the sensitivity of PCR in blood is low due to the presence of amplification inhibitors, and these techniques are most reliable on tissue, sputum, or exudate samples [7,10].

Host-pathogen interaction has been well described and numerous studies contributed to improving the knowledge of host immunity against *Francisella tularensis*. The understanding of the host defense mechanisms in vitro brings light to the risks endured by immunocompromised patients according to the type of immunosuppression. However, to date, available clinical and epidemiological data are insufficient to confirm this in vivo. Indeed, tularemia has been mandatorily notifiable in numerous countries, but there is no specific data on the immunological status of the patients [11]. Therefore, we lack knowledge on the clinical aspects and the burden of tularemia in immunocompromised patients. This is concerning, given the rising development and prescription of immunosuppressive therapies over the past decades, and the recent emergence or re-emergence of tularemia in several European countries [8,12,13,14]. In this article, we first provide an overview on the current knowledge on anti-tularemia immunity and highlight the interplay between immunosuppressive therapies and the host immune responses resulting in a higher susceptibility to *Francisella* in immunocompromised patients. We then review the clinical presentations, diagnostic challenges, and therapeutic approaches of tularemia in immunocompromised patients based on previous studies and case reports. 

### Host Immunity against Francisella

To date, immunity to *Francisella* is not fully understood but the efforts focused on the development of a vaccine over the last decades unraveled the major steps of the host immune response upon *Francisella* infection. In addition to a better understanding of immune deficiency and related diseases, these decades also witnessed the development of immunomodulatory and immunosuppressive therapies. We will discuss the different steps of anti-*Francisella* immune response, and how therapies or illnesses could interfere with these defense mechanisms.

After penetration in the organism, the initial interaction of *Francisella* with host cells occurs through macrophages, dendritic cells, or neutrophils. These host phagocytic cells play an important role in the development of infection through the ability to act as a replicative niche as *Francisella* evades the phagosome within the first hours following phagocytosis to replicate in the cytoplasm [15]. However, even though phagocytosis of *Francisella* is partially impaired, macrophages take a prominent role in anti-*Francisella* immune response. Indeed, within several hours following infection, macrophages are able to produce and release a large amount of pro-inflammatory and Th1-type cytokines, such as tumor necrosis factor α (TNFα), interferon gamma (IFNγ) and interleukin-12 (IL12) [16,17]. Interestingly, mice deficient in TNFα and IFNγ (KO or anti-cytokines monoclonal antibodies) succumbed faster, usually after a sublethal challenge by a vaccine strain with attenuated virulence in humans (live vaccine strain or LVS) of *Francisella tularensis,* suggesting a key role of these cytokines in the initial anti-*Francisella* immune response [18]. Th1-type cytokines, notably IFNγ stimulation, provides resistance to infection and ability to kill the intracellular bacteria to the stimulated macrophages (including alveolar macrophages). This IFNγ stimulation of alveolar macrophages also induces a recruitment of neutrophils to the lungs [19]. The major role played by cytokine production has been extensively reviewed elsewhere [17,20]. Thus, the initial control of infection requires the production of IL12, TNFα, and IFNγ by phagocytic cells. Based on these observations, it seems highly probable that the inhibition of one of these pro-inflammatory cytokines exposes to an increased susceptibility to *Francisella* infection. The progress in immunopathology over the past decades allowed the development of anti-TNFα treatments that provided remarkable therapeutic advances in the management of rheumatologic disorders, such as rheumatoid arthritis and ankylosing spondylitis [21,22]. As expected, these treatments are associated with a higher infectious risk. Concordant with this and with animal models [23], severe cases of tularemia in humans have been described in patients undergoing anti-TNFα therapy [24,25,26].

As macrophages, neutrophils are also capable of early Th1-type pro-inflammatory cytokine production upon *Francisella* infection [27]. These key innate immune cells are known to use production of reactive oxygen species (ROS) via respiratory burst to kill phagocyted pathogens. However, *Francisella* has the ability to inhibit the NADPH oxidase and ROS production in neutrophils in vitro, thus contributing to the development of replicative niches [28,29]. Nonetheless, in vivo, it appears clearly that neutrophil-depleted mice are more susceptible to a normally sublethal LVS challenge, succumb faster, and to a disseminated infection compared to wild type [30,31]. This susceptibility has also been described in humans. Sarria et al. reported a severe and fatal tularemia in a neutropenic patient after a bone marrow transplant [32].

Generation of ROS and reactive nitrogen species (RNS) are known mechanisms elicited by IFNγ that can inhibit the growth of intracellular pathogens. Even though *Francisella* is able to hijack the phagocytic cells degradation mechanisms to replicate in the cytosol, the production of free radicals, notably nitric oxide (NO), and respiratory burst have been described as effective ways of limiting *Francisella* infection [19,20]. Chronic granulomatous disease (CGD) is a hereditary disease caused by a mutation on a NADPH oxidase complex gene. In patients suffering from CGD, the respiratory burst, and thus phagocyte killing of bacteria through ROS is impaired. Hence, patients with CGD are expected to exhibit a higher susceptibility to *Francisella* infection. Maranan et al. reported a case of severe and relapsing Tularemia in a patient with CGD [33]. Furthermore, the association of CGD with *F. philomiragia*, a species genetically related (but epidemiologically distinct) to *F. tularensis* is another argument highlighting the importance of ROS production in controlling *Francisella* infection [34].

Although initial control relies on innate immunity, final clearance of the pathogen is dependent on adaptive immunity that only comes into play several days after the onset of infection [17]. Studies show that mice KO for CD4 and CD8 T-cells, and infected with LVS, do not completely clear the infection and develop a long-term chronic infection [35]. Among these adaptive responses, B cell response and antibody production have been thoroughly studied and proved helpful for the diagnosis of tularemia in humans. Indeed, diagnosis is often based on serological methods as antibodies are detected 2 to 3 weeks after the onset of symptoms [6]. Furthermore, opsonization of bacteria prior to engulfment by phagocytes improves their intracellular destruction, as Fc receptor mediated phagocytosis was associated with major superoxide production, a rapid NADPH dependent oxidative burst, and a restriction of the phagosomal escape [36]. A recent study showed a protective effect of opsonizing antibodies against highly virulent *Francisella tularensis* pulmonary infection in mice depleted in alveolar macrophages [37]. According to this information, patients with B cell depletion might present a higher susceptibility to tularemia.

Although B cells play an important role in adaptive immunity against *Francisella,* T cell mediated response is essential for the control of infection in mammals. Mechanisms by which T cells are involved are not fully elucidated. However, the recruitment of circulating T cells to infected tissues seems to be required for the clearance of *F. tularensis* [38]. It also appears that cytokine production is a key factor of T-mediated response. In mice exposed to a primary sublethal LVS challenge, pulmonary T cells produced type 1 cytokines, such as IFNγ and IL17 [39]. The same Th1 type cytokine production in response to LVS antibody exposure is found in humans [17,40]. Moreover, it has been shown that IFNγ and TNFα contribute to the control of the bacterial growth in macrophage by CD4+ and CD8+ T cells after the initial contact with bacteria [41,42]. Interestingly, the two subsets of T cell respond differently to these factors since CD4+ rely more on IFNγ whereas CD8+ rely almost completely on TNFα [41,43]. Thus, T cells contribute to the control and final clearance of *F*. *tularensis* through a self-sustaining of pro-inflammatory cytokines (IFNγ and TNFα) and mainly through an amplification of the macrophagic response in a cytokine dependent manner. Therefore, we can assess that depletion or functional alteration of T cells by drugs, such as calcineurin inhibitors (CNi), mTOR inhibitors (mTORi), corticosteroids, and others might induce a higher susceptibility to *F. tularensis* in patients undergoing these therapies. A recent review of the immunological targets of immunosuppressive therapies and the related infectious risks has been published by Roberts and Fishman [44]. Among the immunosuppressive therapies, it is interesting to note that CNi, mTORi, and corticosteroids impair both T cell functions and cytokine production, two major tools in the control of *F. tularensis* infection.

## 2. Clinical and Epidemiological Aspects of Tularemia in Immunocompromised

To assess the specific characteristics of this infection in immunocompromised patients, we performed an exhaustive review of the cases reported in the literature using PubMed and Google Scholar with the following research algorithm without date limitation: “(tularemia OR Francisella tularensis) AND (immunocompromised OR Immunodeficient OR transplantation OR immunosuppression)”. Among the 97 articles obtained, we identified 15 case reports or case series with a description of 17 cases of tularemia in immunocompromised patients ranging from 1996 to 2019. The main epidemiological, clinical. and biological characteristics of these cases are presented in Table 1.

### 2.1. Epidemiological Data

Among the 17 cases, 9 were patients from the United States of America, 7 originated from Europe (5 in France, 1 in Germany and 1 in Switzerland) and one was from Turkey. Only 2 were pediatric cases while 15 were adult patients. The median age at diagnosis was 51 years old (range 12–69) and the sex ratio was noticeably in favor of male with only 1 woman out of 17 (6%). The leading cause of immunosuppression was immunosuppressive therapies in 14 patients (82%). Among these patients, seven (41%) were solid organ transplant recipients (SOTR), three (18%) were hematopoietic stem cell transplant (HSCT) recipients, and four (24%) were patients suffering from autoimmune diseases. In addition, two patients were living with acquired immunodeficiency syndrome (AIDS) (12%) and one suffered from CGD (6%).

### 2.2. Clinical and Paraclinical Data

Although clinical presentation might vary, similarities were found in these cases. Among the 17 patients, 16 (94%) presented with fever. General symptoms, such as fatigue and night sweats, were reported in six patients (36%). Respiratory symptoms, including cough and dyspnea with or without respiratory distress, were found in seven patients (41%), whereas abdominal symptoms, including diarrhea and abdominal pain, were found in four (24%). Among the cases reported, five patients (29%) presented with clinically identified enlarged lymph nodes or hepatomegaly. Overall, four patients (24%) presented with glandular or ulceroglandular form while eight (48%) presented with pneumonic form and five (29%) with typhoidal form.

Radiological examinations were performed in 14 patients (82%), 13 of which received thoracic imaging (76%). Interestingly they all showed pathological results with either bilateral (54%) or unilateral (46%) lesions. These lesions included infiltrates (54%), nodules (54%), pleural effusion (23%), mediastinal adenopathy (23%), and one necrotic lesion (7%). Biological samples were drawn from blood or tissues for every patient. Among the 17 patients, 8 (48%) had positive blood culture allowing the recovery of *Francisella tularensis* with a median of 8 days (range 4–21) between sampling and identification. Culture performed on samples other than blood were positive for *F. tularensis* in 5 out of 11 samples corresponding to 5 patients among the 10 (50%) having been sampled. Among these positive cultures, four were on lymph node biopsy and one was on lung tissue biopsy. The characterization of the subspecies was available in 10 cases with 8 subspecies *holarctica* and 2 *tularensis.* When considering the type of samples for which culture remained negative, we found two pleural liquid, one sputum, one bronchoalveolar lavage fluid, one urine, as well as one lung biopsy. Molecular detection of *F. tularensis* with PCR was performed on lymph node biopsy in five patients (29%) with positive result on every sample. Serology was performed on blood in five patients (29%) and was positive in four of them. Histological results on tissue biopsies were provided in only three cases (18%) and always showed epithelioid granuloma with necrosis.

The outcome was favorable in 16 (94%) out of the 17 patients with complete recovery following adequate antimicrobial therapy. Relapse followed by complete recovery with appropriate therapy occurred in 3 (18%) of the 16 patients. Even though the duration of treatment was heterogeneous, similar antimicrobial drugs were used in these patients. Fluoroquinolones were used in 11 patients (64%), 6 of which as a single agent. Among the seven patients (41%) receiving cyclins, four received a monotherapy. Thus, 10 patients (59%) were treated with a monotherapy of either fluoroquinolones or cyclins. Seven patients (41%) received combination therapies combining four different types of antibiotics: fluoroquinolones, cyclins, gentamicin, and penems.

## 3. Discussion

In recent decades, *F. tularensis* has been described as a re-emerging pathogen in Europe [4,11,13,14,54]. The European Food Safety Authority (EFSA) and the European Center for Disease and Prevention Control (ECDC) confirmed a global increase of the animal reservoir [55]. Interestingly animal reservoir and epizootic are strongly associated with human outbreaks of tularemia that often occur within months or year after an expansion of the animal reservoir in specific areas [11,13,14,56]. Moreover, studies showed a positive correlation between climate change and the spread of tularemia among different animal species as it might affect both the hosts and the vectors in terms of survival and activity, which in turn results in the expansion of the animal reservoir [14,57]. Indeed, climate change allows, among other things, the adaptation of disease-carrying animals, such as mosquitoes, biting midges, ticks, rodents, or bats to new geographical areas, which increases the spread of pathogens.

It is in this context that the concept of “One Health” has been developed and is supported by international institutions such as the World Health Organization (WHO), the World Organization for Animal Health (OIE), and the Food and Agriculture Organization (FAO). It promotes the consideration of all the factors involved in the emergence of a disease and requires an effective collaboration between research organizations working in fields such as human health, veterinary health, and environment. This encourages the development and reinforcement of the surveillance networks in addition to a better understanding of the epidemiological characteristics of the disease in animals and in human for the prevention of future outbreaks. 

Several studies evaluated the impact of tularemia in general population at a national level (Table 2) [8,58,59,60,61,62,63]. Median age at diagnosis varied between the different studies. Ulceroglandular and glandular were the predominant forms of tularemia reported in these studies representing from 45% to 72% of the cases [8,58,59,60,61,62]. This is concordant with the historical descriptions of these being the most common forms of tularemia [2,5]. However, in one article reporting cases from one large outbreak in Spain, ulceroglandular and glandular forms represented only 38% of the cases while typhoidal form was the predominant type of tularemia 56% [63]. This exception might be explained by a difference in the definition of the typhoidal form in this study since most of the cases were still handled as outpatients. Among the patients described in our study, 24% presented with ulceroglandular or glandular forms while pneumonic form represented 47% of the cases. Moreover, typhoidal form was found in 29% of the patients. These findings are in contradiction with what was described in the other studies, which exhibited from 0 to 18% of pneumonic forms and from 0 to 20% of typhoidal forms. Turabelidze et al. (CDC report) described 39% of pneumonic forms among their patients. However, typhoidal forms were categorized as pneumonic forms. 

Since pneumonic tularemia mainly results from the inhalation of aerosolized particles but also from hematogenous dissemination, pulmonary involvement either happens as the primary infection or occurs secondary to glandular or typhoidal form. In the general population, clinical manifestation can range from fever with a mild dyspnea or isolated cough to an acute respiratory disorder syndrome. Radiological findings are heterogenous and sometimes represent the only sign of pulmonary involvement [64,65]. When considering the lesions identified on a computed tomography scanner (CT scan), 90% of the patients exhibit enlarged mediastinal lymph nodes or pulmonary infiltrates or nodules [65]. These findings are concordant with the description of the pulmonary lesions identified in FDG-positron emission tomography where hypermetabolic lymph nodes are observed in 100% of the cases and pulmonary lesion and nodules in 74% [66]. However, the radiological diagnosis is complicated and often misleading as these lesions are not distinguishable from those of lung cancers or hematological malignancies [66]. 

Even though the clinical presentations of pneumonic tularemia in the immunocompromised patients described in this study are concordant with the description of this form in the general population, these results seem to highlight a different pattern in immunocompromised patients with preponderant pneumonic and typhoidal forms compared to the general population more frequently presenting with ulceroglandular or glandular forms. This predominance of the pneumonic form suggests that contaminations of immunocompromised patients mainly occur through inhalation even though secondary lung involvement due to hematogenous dissemination remains a possibility. There is no data providing a clear explanation for this pulmonary involvement on a cellular or molecular level. However, as stated above, immunosuppressive therapies impair the ability of the host immune system to control the infection. It appears highly probable that after the first contact with the bacteria, this lack of control leads to a facilitated parenchymal proliferation and dissemination. Interestingly, Roberts et al. observed that pulmonary T cells were inadequate for the complete control of tularemia infection in mice and that a recruitment of circulating T cells was required for the clearance of the bacteria from the lungs and to prevent the dissemination [38]. Hence, lymphopenia or functional alteration of lymphocytes induced by immunosuppressive therapies or diseases might favor parenchymal proliferation and dissemination from the lungs, even with a lower inoculum, and are possible explanations to the particular clinical pattern observed in this study.

To date, there is no gold standard for the diagnosis of tularemia and it still mainly relies on serological tests [6]. The isolation and identification of *F. tularensis* by culture are fastidious and result with less than 10% of positive cultures in immunocompetent patient [4,8,10]. However, PCR based methods can be useful and provide an early diagnosis when performed on tissue, skin ulcer, and other type of biological samples [7,10]. In this study, 48% of the patients had positive blood cultures for *F. tularensis*. Interestingly, it has been previously reported that most of the cases of *F. tularensis* bacteremia were associated with pneumonic tularemia, which is consistent with our results [67]. This also highlights the severity of tularemia in immunocompromised patients, as bacteremia is associated with more pejorative outcomes [67,68]. Serological testing and PCR were both performed on 29% of the patients with positive results in 80% and 100%, respectively. These findings might suggest that the diagnosis was rarely suspected initially. However, when performed, these tests allowed the confirmation of the diagnosis. This emphasizes on the importance of a better knowledge of the characteristics of tularemia for physicians to suspect this diagnosis and set up the most relevant diagnostic strategy in concerned patients. 

The treatment of tularemia relies on antimicrobial therapy with quinolones, cyclins, and aminoglycosides as the main class of antibiotics recommended. In this study, patients mainly received either one or a combination of these with a favorable outcome in 94% of the cases. Thus, we can expect a similarity in terms of susceptibility to antibiotics between immunocompromised patients and general population with respect of the heterogeneity of the treatment durations presented in our study. 

In conclusion, we can expect, in future years, a persistent increase of the animal reservoir related to global warming. This includes a rise of hosts within a wide range of species, but also of the vectors, as it has already been witnessed for other infectious diseases in humans or animals (e.g., dengue virus, Lyme disease, blue tongue virus, etc.). Moreover, the development of novel immunosuppressive therapies will lead to an increasing number of immunocompromised patients. Thus, it appears important to highlight the characteristics of tularemia in immunocompromised patients. 

We identified a more frequent pulmonary involvement among immunocompromised patients presenting tularemia. Diagnosis is often tedious with nonspecific clinical symptoms and radiological findings exhibiting pulmonary lesions or mediastinal adenopathy indistinguishable from malignancies. In this context, PCR-based methods are useful and allow a faster diagnosis than serological tests. Thus, tularemia should be suspected in immunocompromised patients presenting with fever and respiratory symptoms or a history of potential exposure. 

The pneumonic presentation suggests more frequent contamination through inhalation, emphasizing the importance of preventive measures in immunocompromised patients in areas of high risk of tularemia, such as the use of surgical masks in outdoor activities, and avoiding contact with dead animals. The monitoring of tularemia in wildlife via surveillance networks should be encouraged as it allows the identification of areas with high risks of outbreaks. 

## Figures and Tables

**Table 1:** Cases of tularemia in immunocompromised patients from litterature.

Age Country	Gender	Year	Pathology / Immunosuppressive Therapy	Main Symptoms	Imaging Results	Biological Results	Treatment	Outcome	Author
12 USA	M	1996	AIDS: CD4 0/mm3	Fever; nausea; headaches; photophobia without meningismus; abdominal pain with hepatosplenomegaly; cough; tachypnea	*Chest radiograph:* left lower lobe infiltrate	Blood cultures: positives for *F. tularensis* after 13 days. Tularemia Serology: negative	Ceftazidime + Vancomycin IV 10 days Gentamicin + ampicillin IV 7 days first relapse Gentamicin 10 days then tetracycline 14 days second relapse tetracycline 21 days	Complete recovery (after 2 relapses)	[45]
14 USA	M	1997	Chronic granulomatous disease	Fever; unproductive cough; recurring after treatment and lobectomy	*Chest radiograph*: left lower-lobe infiltrate with pleural effusion. After 3 months *Chest CT scan*: necrotic area within the left lower lobe	Pleural and lung culture: negative Tularemia serology: positive	Doxycycline 7 days relapse Doxycycline 14 days Lower left lobe Lobectomy relapse Gentamicin + Ticar/clav IV 21 days + Doxy 30 days	Complete recovery (after 2 relapses)	[33]
50 USA	M	1999	Liver transplant: Prednisolone, 10 mg/day Azathioprine, 75 mg/day	Fever, arthromyalgia, and pneumonia	*Chest radiograph*: right middle lobe infiltrate	Bronchoalveolar lavage fluid testing: negative Blood cultures: positives for *F. tularensis* subsp. *holarctica* after 9 days.	Levofloxacin: 500 mg/day, 21 days	Complete recovery (no relapse)	[46]
33 USA	M	1999	AIDS: CD4 220/mm3	Fever; dry cough; headache; myalgia; pneumonia and no modification of the previous lymphadenopathies	*Chest radiograph*: ill-defined bibasilar abnormalities	Blood cultures: positive for *F. tularensis* subsp. *holarctica* after 21 days. Urine and Sputum cultures: negative	Levofloxacin: 500 mg/day, 10 days	Complete recovery (no relapse)	[46]
61 USA	M	1999	7 months after peripheral blood stem cell transplantation for acute myeloid leukemia (AML) conditioning: busulfan + cyclophosphamide	Fever, chills and fatigue	*Chest CT scan*: right lower lobe nodule	Culture of nodule needle aspiration: positive for *F. tularensis* after 6 days	Imipenem IV 7 days then Ciprofloxacin 750 mg b.i.d 28 days	Complete recovery (no relapse)	[47]
43 USA	M	2003	Chemotherapy followed by bone marrow transplant for ALL Conditioning not precised	Fever, lethargy, inguinal lymph nodes expansion	none	Blood cultures positives after 4 days, identification of *F. tularensis subsp. tularensis* post mortem	Imipenem + vancomycin, 12 days with Gentamicin 5 days	Deceased (on d14 of symptoms)	[32]
69 USA	M	2004	Kidney transplant: mycophenolate mofetil rapamycin prednisone	Fever, chills, fatigue, vomiting, diarrhea	*Chest radiograph*: patchy infiltrate in the left lung	Blood culture positive for *F. tularensis* subsp. *tularensis* after 7 days	Doxycycline for 14 days	Complete recovery (no relapse)	[48]
59 USA	M	2005	11 years post kidney transplant: prednisone; mycophenolate mofetil; cyclosporine	Persistent fever	*Chest CT-scan*: multiple pulmonary nodules	Nodule biopsy cultures: positive for *F. tularensis*	Fluoroquinolone (dosage and duration unknown)	Clinical improvement	[49]
58 France	M	2009	Rheumatoid arthritis: methotrexate + adalimumab	Fever, plaque on the left leg with central necrotic area, enlarged left inguinal lymph node with skin fistula	None	Skin biopsy histopathology: epithelioid granulomas with giant cells and central necrosis. Tularemia serology: positive PCR for *F. tularensis:* positive on a lymph node biopsy	Doxycycline for 6 weeks	Complete recovery (no relapse)	[24]
54 Germany	M	2010	4 years after stem cell transplant for AML. With chronic graft-versus-host-disease: tacrolimus + steroids	Fever, chills, dyspnea	CT scan: large infiltrate in the right upper lobe	Blood culture: positive for *F. tularensis subsp. holarctica* after 7 days	Imipenem + levofloxacin for 8 days + Doxycycline for 8 days	Complete recovery	[50]
69 France	M	2010	15 years post kidney transplant: prednisolone; mycophenolate mofetil; cyclosporine a	Fever, chills, cough and sputum	*Chest radiograph*: bilateral interstitial infiltrates	Blood culture: positive for *F. tularensis* after 10 days. PCR on cultured colony: positive for *F. tularensis subsp. holarctica*	Ciprofloxacin 500 mg/day (adapted to renal function) for 14 days	Complete recovery (no relapse)	[51]
24 Turkey	M	2012	12 months after kidney transplant. prednisolone; mycophenolate mofetil; tacrolimus	Cervical lymphadenopathy	none	Lymph node biopsy: chronic necrotizing granulomatous inflammation Real-time PCR–for tularemia on lymph node: positive. Serology: positive	Doxycycline for 10 days	Complete recovery (no relapse)	[52]
32 France	W	2014	Severe psoriatic arthritis: certolizumab; methotrexate	Fever, right elbow pain with functional impairment.	*Initial Elbow CT scan*: large collection in the right elbow. *Second CT scan:* communicating axillary collections compatible with necrotic confluent adenopathy	Glandular abscess aspirate culture: positive after 4 days. *F. tularensis* subsp. *holarctica* identified after amplification and sequencing of 16SrDNA	Ciprofloxacin + gentamicin for 14 days; then ciprofloxacin for 14 days relapse; ciprofloxacin + doxycycline for 4 months	Complete recovery (after 1 relapse)	[26]
51 France	M	2015	7 years after liver transplant: tacrolimus mycophenolate mofetil	Septic shock, acute respiratory distress syndrome, ketoacidosis,	*Chest radiograph*: bilateral alveolar opacities *Thoracic CT-Scan*: mediastinal lymphadenopathies and bilateral nodular lesions	Blood culture: positive after 5 days. Strain unidentified Amplification and sequencing allowed identification of *Francisella tularensis* subsp. *holartica*	Ciprofloxacin 500 mg b.i.d for 14 days	Complete recovery (no relapse)	[9]
64 France	M	2016	4 Years after heart transplantation: prednisolone cyclosporin mycophenolate mofetil	Fever, chills, night sweats, unproductive cough, progressive respiratory distress	*CT-scan:* pleural effusion and mediastinal lymphadenopathies *PET-scan:* hypermetabolism of mediastinal and celiacomesenteric lymphadenopathies and pulmonary parenchymatous lesions	Pleural liquid cultures: negative. PCR *F. tularensis* positive on two lymph node biopsies. Culture of lymph node biopsy: positive for *Francisella* *tularensis* subsp. *holarctica*	Ciprofloxacin 750 mg b.i.d. + gentamicin 300 mg for 7 days; then ciprofloxacin for 14 days	Complete recovery (no relapse)	[9]
51 USA	M	2017	Rheumatoid arthritis: infliximab, leflunomide	Fever, fatigue, diarrhea, chest pain, confusion	*CT scan*: multiple pulmonary parenchymal nodules with mediastinal adenopathy and a right pleural effusion	Lung biopsy culture: positive for *Francisella tularensis*	Intravenous infusion of gentamicin and oral ciprofloxacin	Complete recovery (no relapse)	[24]
25 Switzerland	M	2019	Psoriasis adalimumab	Fever, chills, weight loss, night sweats, diarrhea, dysuria, headaches, painful neck lymph node	Chest CT scan: mass near the right hilus, infiltrations in the left and right upper lung lobe, mediastinal lymphadenopathy	Blood cultures: negative lymph nodes biopsy: central necrotizing epithelioid cell granulomas PCR of the biopsy was positive for F. tularensis ssp. Holarctica serology: positive	Ciprofloxacin 750 mg bid. For 18 days	Complete recovery	[53]

**Table 2:** Comparison of the clinical presentations of tularemia in different studies.

	Darmon Cuti et al. [8]	Udurgucu et al. [58]	Appelt et al. [59]	Mailles et al. [60]	Pérez- Castrillon et al. [61]	Turabelidze et al. [62]	Martín et al. [63]	Present Study
Pneumonic form	18%	0%	12.1%	10%	3.5%	39% (combined)	7.7%	47%
Typhoidal form	7.9%	0%	1.1%	10%	20.4%		56.6%	29%
Ulceroglandular form	34.5%	1.9%	15.6%	26%	61.3%	42%	16%	24% (combined)
Glandular form	27%	62.3%	29.7%	46%	9.2%	16%	12.1%	
Other forms	12.6%	35.8% (oropharyngeal)	17.7%	8%	5.6%	3%	7.6%	0%

## Data Availability

Data supporting the results are available in the original articles cited in the reference section.
